# Editorial: Cellular nanomotion as a new signature of life

**DOI:** 10.3389/fmicb.2024.1390002

**Published:** 2024-03-11

**Authors:** Ronnie G. Willaert, Sandor Kasas

**Affiliations:** ^1^Research Group Structural Biology Brussels, Alliance Research Group VUB-UGhent NanoMicrobiology (NAMI), Vrije Universiteit Brussel (VUB), Brussels, Belgium; ^2^International Joint Research Group VUB-EPFL BioNanotechnology & NanoMedicine (NANO), Vrije Universiteit Brussel (VUB), Brussels, Belgium; ^3^Laboratory of Biological Electron Microscopy, Ecole Polytechnique Fédérale de Lausanne (EPFL) and University of Lausanne, Lausanne, Switzerland; ^4^Centre Universitaire Romand de Médecine Légale, UFAM, Université de Lausanne, Lausanne, Switzerland

**Keywords:** cellular nanomotion, AFM-based nanomotion, optical nanomotion, bacteria, yeasts, erythrocytes, neutrophils, mitochondria

Life can be characterized among others by the ability of the organisms to move. Recently, it has been known that the vast majority of all living organisms on Earth oscillate at a nanometric scale. These observations were made possible by the use of atomic force microscopes (AFM) since they are very sensitive displacement detectors and can detect displacements down to 0.1 Å. This instrument highlighted for the very first time that cellular nanomotions last as long as the organism is alive and cease upon death.

The discovery of cellular nanomotion permitted the development of label-free, rapid antibiotic, antifungal, and antimitotic sensitivity testing. The methodology consists of attaching the cells to an AFM cantilever, exposing them to various antimicrobial, antifungal, or antimitotic drugs, and monitoring the cantilever oscillations as a function of time. Cellular nanomotion not only indicated the life or death state of microorganisms but also provided insights into their metabolism and, in some cases, their virulence.

Cellular nanomation can also be detected by other methods such as nanodrums, plasmonic imaging, the phase noise of a resonant crystal, total internal reflection microscopy (TIRM), intrinsic phase-shift spectroscopy, or a basic optical microscope. Optical nanomotion detection (ONMD) is the technique that uses a classical optical microscope equipped with a video camera to measure the nanoscale oscillations or vibrations of cells.

Cellular nanomotion is a new field of research that is expected to provide interesting new fundamental biological insights into cells. In this Research Topic “*Cellular nanomotion as a signature of life*,” the most recent developments in AFM-cantilever and optical nanomotion detection of cellular nanomotion are presented. The cellular nanomotion studies involved bacteria, mitochondria, yeasts, and mammalian cells. In addition, a new algorithm to process ONMD data was presented.

Pleskova et al. used AFM to study the nanomotion of different clinical strains of motile and non-motile bacteria. They highlighted distinct nanomotion patterns between different bacteria. Flagellated bacteria (*Escherichia coli, Proteus mirabilis*) showed larger nanomotion than the non-flagellated ones (*Staphylococcus aureus, Klebsiella pneumoniae*). By depositing neutrophil granulocytes on AFM-cantilevers, a significant increase of nanomotion was observed upon phagocytosis.

Girasole et al. also employed AFM to study human red blood cells (RBCs) in patients with favism, a genetic defect of erythrocytes that affects their ability to respond to oxidative stresses but also determines differences in the metabolic and structural characteristics of the cells. They showed that RBCs from favism patients exhibit a prolonged nanomotion activity to the forced activation of the ATP synthesis compared to healthy cells. They also highlighted that the favism cells showed a greater resilience to the aging related insults.

Starodubtseva et al. used ONMD to assess the effect of X-ray radiation on the opportunistic pathogenic yeast *Candida albicans* and its sensitivity to antifungal drugs. They demonstrated that exposure to X-ray radiation and radiation in combination with the antifungal fluconazole compromised low-frequency nanomotion. The nanomotion rate was found to depend on the phase of the cell cycle, the absorbed radiation dose, the fluconazole concentration, and the duration of the post-irradiation period.

Parmar et al. demonstrated that sub-cellular organelles, such as mitochondria, could also be detected by a basic optical microscope. The study involves the OMND monitoring of mitochondria exposed to mitochondrial toxins, citric acid cycle intermediates, and dietary and bacterial fermentation products (short-chain fatty acids) at various doses and durations. The study demonstrates that ONMD monitoring of mitochondria had several advantages as compared to the classical methods since it was rapid, had single organelle sensitivity, and was label- and attachment-free.

Villalba et al. presented a novel algorithm to process ONMD data sets. The method was applied to motile (*E. coli*), non-motile (*S. aureus*), rapid (*E. coli*), and slow growing (*Mycobacterium smegmatis*) bacteria as well as different yeasts (*C. albicans, Saccharomyces cerevisiae*). The method enables the analysis of a whole population of microorganisms without tracking individual cells. It permitted the acceleration of the data processing procedure and reduced the complexity of the data processing algorithm.

The initial applications of cellular nanomotion studies were focused on life-death transitions, i.e., the development of rapid antibiotic and antifungal sensitivity tests. Two contributions highlight that cellular nanomotion studies can be extended to assess the effect of radiation and the data processing of life-dead transitions can be significantly simplified using a new nanomotion detection algorithm. Three contributions extended nanomotion analysis to detect metabolic changes occurring in red blood cells, neutrophiles, and mitochondria. The study on mitochondria demonstrates that ONMD can be used to monitor single, isolated organelles too. These studies pave the way to employ nanomotion detection as a label-free, rapid, and simple diagnosis tool.

## Author contributions

RW: Writing – original draft, Writing – review & editing. SK: Writing – original draft, Writing – review & editing.

